# Supplementary material for: Baicalin-Copper Complex Modulates Gut Microbiota, Inflammatory Responses, and Hormone Secretion in DON-Challenged Piglets
Source: Animals (Basel). 2020 Aug 31;10(9):1535. doi: 10.3390/ani10091535 (PMC7552336; doi:10.3390/ani10091535)
Supplement: Supplementary file 1 [file animals-10-01535-s001.pdf]

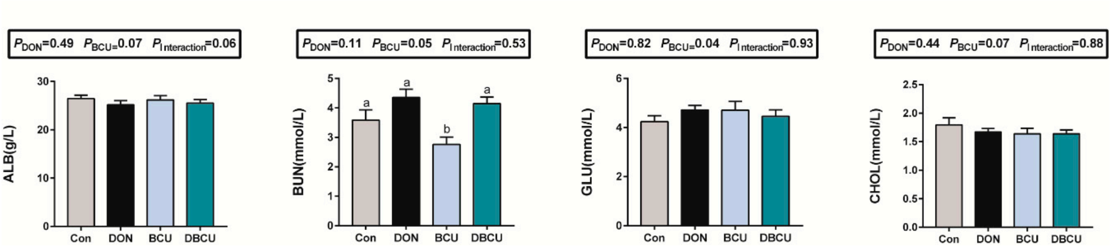

Figure S1. Effects of dietary DON and BCU on serum Biochemical Indexes in piglets. Values are means ± SEMs, n = 7. Different letters in the picture represent differences, $P < 0.05$. Basal diet (Con); 4 mg DON/kg diet (DON); 5 g BCU/kg diet (BCU); 5 g BCU/kg with 4 mg DON/kg diet (DBCU). ALB: Albumin; BUN: Blood urea nitrogen; CHOL: Total cholesterol; GLU: Glucose.

Table S1. Primers used for Real-time q-PCR.

Gene	Primer sequence (5'-3')	Accession number	Size (bp)	Tm (℃)
PYY	AGATATGCTAATACACCGAT<br>CCAAACCCTTCTCAGATG	XM_021066092.1	93	60.00
SST	CTCTCCATCGTCCTGGCTCT<br>GTTCTCTGTCTGGTTGGGTTCAG	NM_001009583.1	159	60.00
INR	GGCATGGTGTACGAGGGAAA<br>AGGCCTCGTTGAGAAACTCG	XM_021083943.1	124	60.00
CCK-1R	GTGGTCCACAGCCTTCTTAT<br>TCATTTTCGATCCCCAGTT	XM_021101084.1	68	60.00
CCK-2R	GCGGCGATCTTTCTGATGAG<br>GCAGGAAGGCGTTGGTGA	XM_021062350.1	97	60.00
GLP-1R	TACTTCTGGCTGCTGGTGGAG<br>ACCCCAGCCTATGCTCAGGTA	NM_001256594.1	105	60.00
GLP-2R	TGTCCTACGTGTCGGAGATGTC<br>TAATTGGCGCCCACGAA	XM_021066117.1	76	60.00
c-Fos	CGTGGAGCCAGTCAAGAAC<br>CTCCCAGTCTGCTGCATAG	NM_001123113.1	157	60.00
AGRP	GCAGGCCGAGGCCAA<br>CGTGCCTTGCGTCCTTC	XM_021093546.1	57	60.00
NPY	TCGGCGTTGAGACATTACATCA<br>GTCTCGGGACTAGATCGTTTTCC	NM_001256367.1	68	60.00
POMC	TGCTTGGAAGATGCCGAGAT<br>GCGGAGAGATCTGGTTTGCA	NM_213858.1	177	60.00
5-HT	ACAGGAACAAGATGACCCCT<br>AGGAGGAACGGGATGTAGAA	NM_001001267.1	277	60.00
AKT	TGTGGCAGGATGTGTATGAGA<br>GTAGGAGAACTGGGGGAAGTG	XM_021081501.1	188	60.00
HTR3A 1	GCCCTTCTGGTGATCAGCTT<br>AGCAGTCATCGGTCTTGGTG	XM_003357301.4	196	60.00
HTR3A 2	CCCCCAGCCTTGCTTTTAGA<br>AGGGAAGTGGCCATAGGTGA	XM_003357301.4	122	60.00
HTR3B 1	GTGTGCTCCACGAGTCTTCA<br>GGGTGACTACAACCAGAGGC	XM_021062866.1	98	60.00
HTR3B 2	AGAGCAGCGCTGGAGATTTT<br>GGCTCACAACATAGGCCAGT	XM_021062866.1	78	60.00
COX-2	AAGCGAGGACCAGCTTTCACCAAA<br>GCGCAGTTTATGCTGTCTCTCCAA	NM_214321.1	93	60.00
β-actin	CACGCCATCCTGCGTCTGGA<br>AGCACCGTGTTGGCGTAGAG	XM_021086047.1	380	60.00
GAPDH	ACACTCACTCTTCTACCTTTG<br>CAAATTCATTGTCGTACCAG	XM_021091114.1	90	60.00
